# The Use of Phage Cocktail and Various Antibacterial Agents in Combination to Prevent the Emergence of Phage Resistance

**DOI:** 10.3390/antibiotics12061077

**Published:** 2023-06-20

**Authors:** Hoang Minh Duc, Yu Zhang, Son Minh Hoang, Yoshimitsu Masuda, Ken-Ichi Honjoh, Takahisa Miyamoto

**Affiliations:** 1 Department of Bioscience and Biotechnology, Graduate School of Bioresource and Bioenvironmental Sciences, Kyushu University, 744 Motooka, Nishi-ku, Fukuoka 819-0395, Japan; 2 Department of Veterinary Public Health, Faculty of Veterinary Medicine, Vietnam National University of Agriculture, Hanoi 12400, Vietnam; 3 State Key Laboratory of Microbial Resources, Institute of Microbiology, Chinese Academy of Sciences, Beijing 100101, China; 4 Department of Histology and Embryo, Faculty of Veterinary Medicine, Vietnam National University of Agriculture, Hanoi 12400, Vietnam

**Keywords:** bacteriophages, EDTA, nisin, polylysine, biocontrol, bacterial cocktail

## Abstract

Bacterial food poisoning cases due to *Salmonella* and *E. coli* O157:H7 have been linked with the consumption of a variety of food products, threatening public health around the world. This study describes the combined effects of a phage cocktail (STG2, SEG5, and PS5), EDTA, nisin, and polylysine against the bacterial cocktail consisting of *S. typhimurium*, *S. enteritidis*, and *E. coli* O157:H7. Overall, phage cocktail (alone or in combination with nisin or/and polylysine) not only showed great antibacterial effects against bacterial cocktail at different temperatures (4 °C, 24 °C, and 37 °C), but also totally inhibited the emergence of phage resistance during the incubation period. These results suggest that the combination of phages with nisin or/and polylysine has great potential to simultaneously control *S. typhimurium*, *S. enteritidis*, and *E. coli* O157:H7.

## 1. Introduction

Bacterial food poisoning is a global public health concern, affecting millions of people every year around the world. *Salmonella* is one of the major causes of foodborne illnesses and includes more than 2500 serotypes, of which *S. typhimurium* and *S. enteritidis* are two major serovars frequently related to human infection [1]. In the United States, about 1.35 million infections, 26,500 hospitalizations, and 420 deaths annually are caused by *Salmonella* spp. mainly found in food products, such as poultry and eggs [2]. *Escherichia coli* O157:H7 has also been identified as another important foodborne pathogen linked to the consumption of fresh produce, which could produce shiga-like toxins and cause severe symptoms, including mild diarrhea, hemorrhagic colitis, hemolytic uremic syndrome, and thrombotic thrombocytopenic purpura [3,4]. Every year, there are more than 1 million illnesses, 128 deaths, and nearly 13,000 disability-adjusted life years (DALYs) as a result of *E. coli* O157:H7 infection [5]. Therefore, reducing these contaminations associated with food is essential in order to protect human health.

The use of bacteriophages, which are bacterial viruses with high antibacterial efficacy and specificity to the target bacteria, as well as other favorable features such as being harmless to humans and abundant in the environment, has been increasingly recognized as a promising disinfection approach in the food industry [6,7,8]. Over the past two decades, there are numerous studies which suggest the potential use of phages as biocontrol strategies against a variety of bacterial contaminations [1,3,6,9,10]. Unfortunately, phage therapy still faces some challenging issues, e.g., bacteria easily evolve resistance to phage infection resulting in the failure of phage treatment [10,11]. To overcome this problem, the use of phage cocktails over a single phage is often encouraged for phage therapy, which could not only constrain the emergence of phage-resistant bacterial mutants but also broaden antibacterial spectrum activity [1,4,7,12]. Although it cannot always inhibit the regrowth of phage-resistant bacteria, studies have repeatedly shown that the emergence of resistance to phage cocktails is less common than an individual phage [1,4,7,12].

Compared to phages, other common food additives usually have a broader spectrum against pathogens with different antibacterial mechanisms [13,14]. Among these food additives, ethylenediaminetetraacetic acid (EDTA) has drawn attention, and it has been approved to use in a variety of food products by the World Health Organization (WHO) and the Food and Drug Association (FDA) [15]. EDTA damages the outer membrane of Gram-negative bacteria by chelating divalent cations in the lipopolysaccharide (LPS), thus increasing the permeability of bacterial cells to other antibacterials [16]. Nisin, a class I bacteriocin, is another famous food additive capable of inhibiting the growth of food-spoilage bacteria and foodborne pathogens, especially Gram-positive bacteria, which could be used alone or in combination with other preservatives, or also with several physical treatments [10,14]. As another common food additive, ε-polylysine is a cationic, naturally occurring polypeptide consisting of L-lysine units that are biodegradable, water-soluble, nontoxic, and edible [17,18]. The antibacterial characteristics of ε-polylysine are well established; thus, it has been increasingly used as a food preservative in recent years [19].

Although there are some previous studies on the combination of a phage with food additives as biocontrol strategies against foodborne pathogens, most of them mainly focus on a single pathogen instead of a bacterial mixture [10,12]. It is well known that bacteria, in nature, particularly foodstuff, frequently coexist in multispecies communities [20], making them less susceptible to antibacterial agents and posing a serious threat to food safety. Therefore, the objective of this study was to evaluate the potential use of phage cocktail combined with several food additives including EDTA, nisin, and polylysine to fight against a bacterial mixture of *S. typhimurium*, *S. enteritidis*, and *E. coli* O157:H7. This is the first report on the combined effect of phage cocktail with EDTA, nisin, and polylysine against a bacterial mixture of *S. typhimurium*, *S. enteritidis*, and *E. coli* O157:H7.

## 2. Results

### 2.1. The Combined Effect of Phages and EDTA on the Viability of Bacterial Cocktail

**Figure 1:**
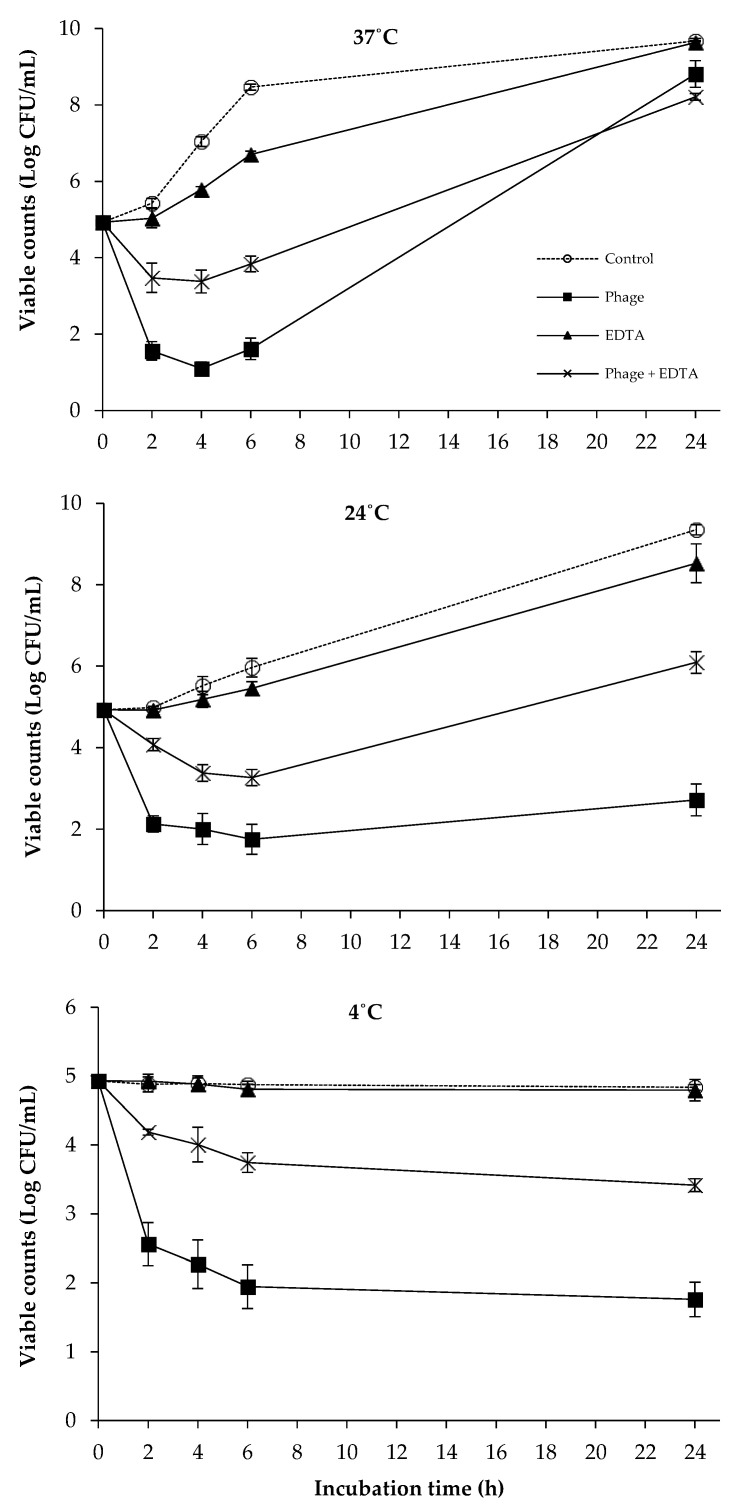
The combined effect of phages and EDTA on the viability of bacterial cocktail at 37 °C, 24 °C, and 4 °C.

Overall, treatments with phages or/and EDTA caused significant reductions in the bacterial viability of *S. enteritidis, S. typhimurium*, and *E. coli* O157:H7 in LB broth compared to those of phages and EDTA-free controls at 37 °C, 24 °C, and 4 °C (Figure 1). In the controls, viable counts of bacterial cocktail gradually increased and reached a plateau after 24 h at 37 °C and 24 °C. While the bacteria did not grow during the whole incubation period at 4 °C, steady viable counts were maintained and similar to the inoculum level. The addition of EDTA alone only caused slight reductions in the bacterial viability at both 37 °C and 24 °C compared to the controls, and obvious changes were not observed at 4 °C. For the phage treatment, the viable counts dramatically decreased by at least more than 2 log after 2 h of incubation at all the three temperatures tested when compared to controls. The significant bacterial regrowth was not observed in phage treatments performed at 24 °C and 4 °C, and there was at least a three-log reduction at the end of experiment time (24 h). However, at 37 °C, the bacterial counts in the phage treatment reached a level close to that in the control after 24 h of incubation. In general, the combined treatment of phage and EDTA did not show a stronger antibacterial efficacy against the bacterial cocktail of *S. enteritidis*, *S. typhimurium*, and *E. coli* O157:H7 in comparison with phage and EDTA alone in all the tested conditions.

### 2.2. The Combined Effect of Phages and Nisin on the Viability of Bacterial Cocktail

**Figure 2:**
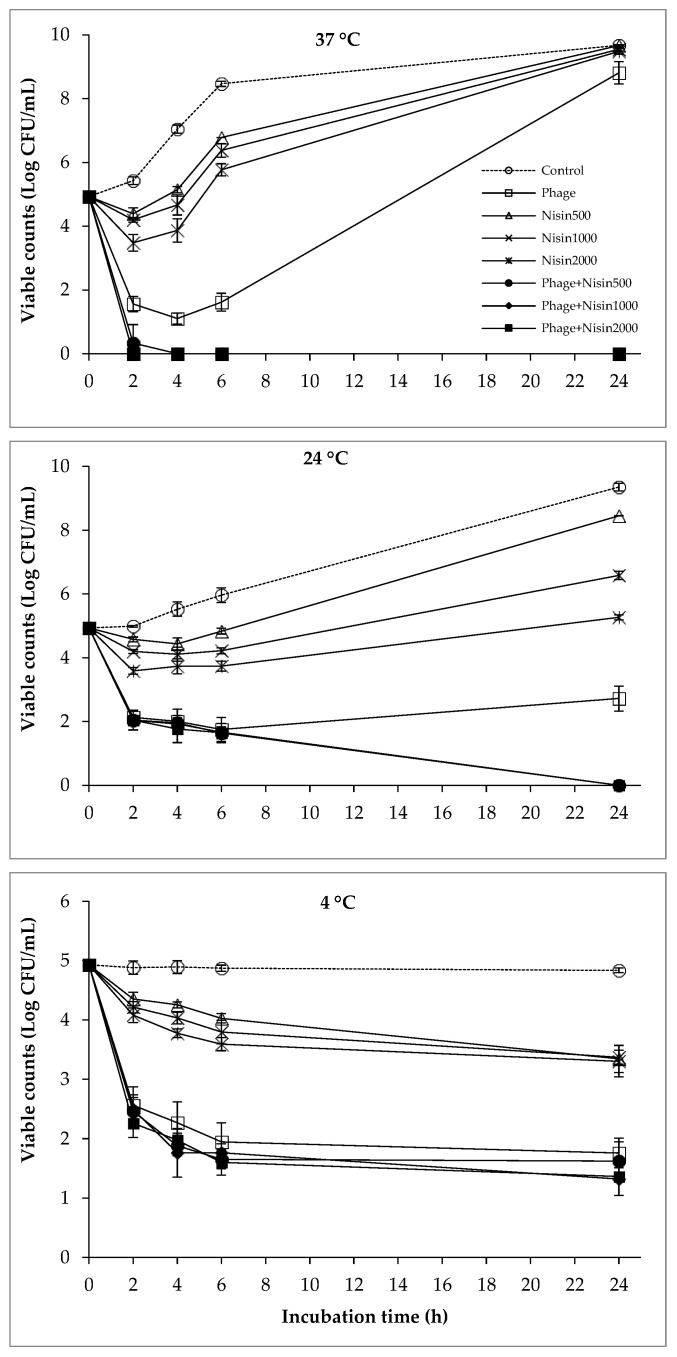
The combined effect of phages and nisin on the viability of bacterial cocktail at 37 °C, 24 °C, and 4 °C.

The efficacy of phages and nisin (alone or in combination) against the cocktail of *S. enteritidis*, *S. typhimurium*, and *E. coli* O157:H7 was also tested (Figure 2). The combination of phages and nisin was shown to be more effective than phages and nisin alone during all the tested temperatures, and the antibacterial activity of nisin increased with increasing concentrations. In the experiment conducted at 37 °C, the combined effects of phages and nisin were observed, and their combination reduced the bacterial viability to below the detection limit at 4 h, and this effect was maintained over 24 h. In contrast, the treatment of phages or nisin alone could not prevent the regrowth of bacteria and viable counts of bacterial cocktail in the treatment was close to that in the control at the end of the incubation time. At 24 °C, the combined treatment of phages and nisin was also able to decrease the viable bacterial cocktail counts to under the detection limit, but only after 24 h of incubation. On the contrary, the bacteria recovery was observed at 24 h in the treatment of phages or nisin alone at 24 °C. The combined effect of phages and nisin was not obvious at 4 °C, and the bacterial viability in phages and nisin combination was relatively similar to those of phages alone.

### 2.3. The Combined Effect of Nisin and EDTA on the Viability of Bacterial Cocktail 

**Figure 3:**
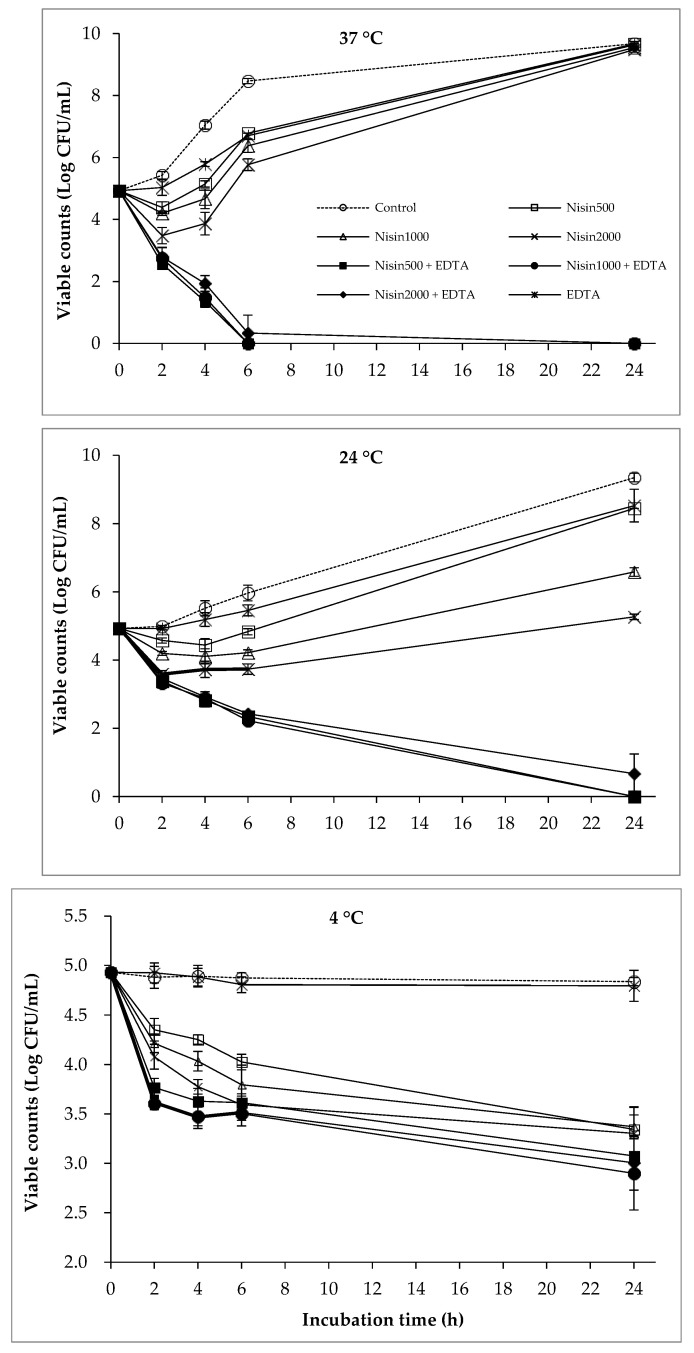
The combined effect of nisin and EDTA on the viability of bacterial cocktail at 37 °C, 24 °C, and 4 °C.

The antibacterial activity of EDTA, nisin, and their combination against the bacterial cocktail at various temperatures is shown in Figure 3. The combined effect of nisin and EDTA was observed at both 37 °C and 24 °C. At the end of the incubation time, the bacterial regrowth did not occur in the nisin+EDTA treatment. On the contrary, the bacterial regrowth was recorded in nisin and EDTA treatment alone after 2 h of incubation at 37 °C and 24 °C. Although the combined treatment of nisin and EDTA showed a little combined effect at 4 °C, it was still more effective than single treatments and decreased the viable counts by around two logs at the end of the incubation period.

### 2.4. The Combined Effect of Phages, Nisin, and EDTA on the Viability of Bacterial Cocktail 

**Figure 4:**
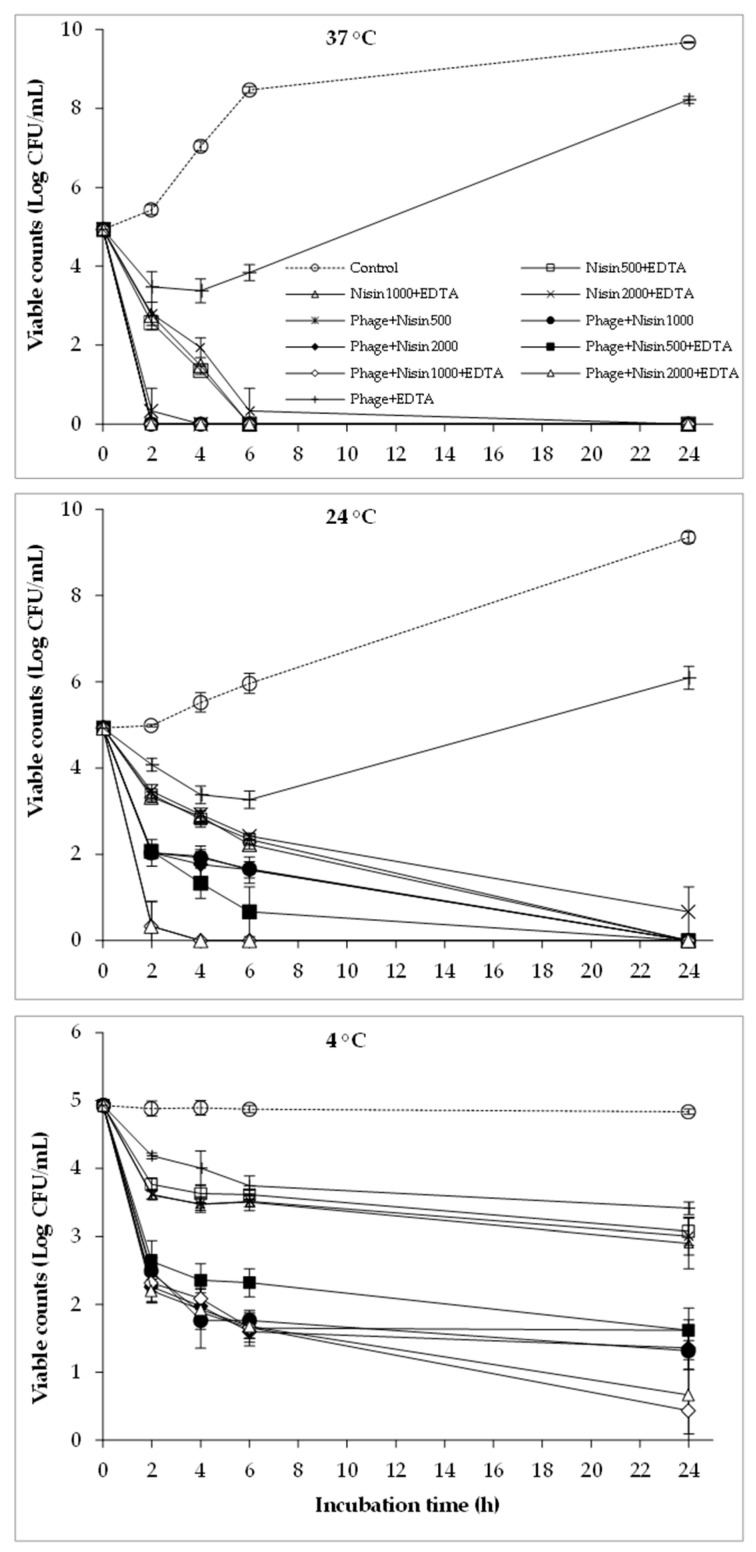
The combined effect of phages, nisin, and EDTA on the viability of bacterial cocktail at 37 °C, 24 °C, and 4 °C.

Figure 4 reveals the efficacy of the co-treatment of phages with nisin and EDTA against the bacterial cocktail. The viable bacterial counts were dramatically decreased by this combination at all the three temperatures tested. The highest reductions were obtained at 37 °C by applying phage+nisin500/1000/2000+EDTA, in turn decreasing the bacterial counts to lower than detection limit within 2 h. Compared to 37 °C, the application of phage+nisin1000/2000+EDTA at 24 °C showed relatively similar effects, while phage+nisin500+EDTA displayed weaker antibacterial activity. At 4 °C, although the phage+nisin+EDTA combination did not reduce the viable counts to under the detection limit, it still produced additional reductions compared to phage+nisin and nisin+EDTA. Notably, bacterial regrowth was not observed in the treatments with phage, nisin, and EDTA in combinations under all temperatures tested, except for the phage+EDTA combination.

### 2.5. The Combined Effect of Phages, Nisin, and Polylysine on the Viability of Bacterial Cocktail

**Figure 5:**
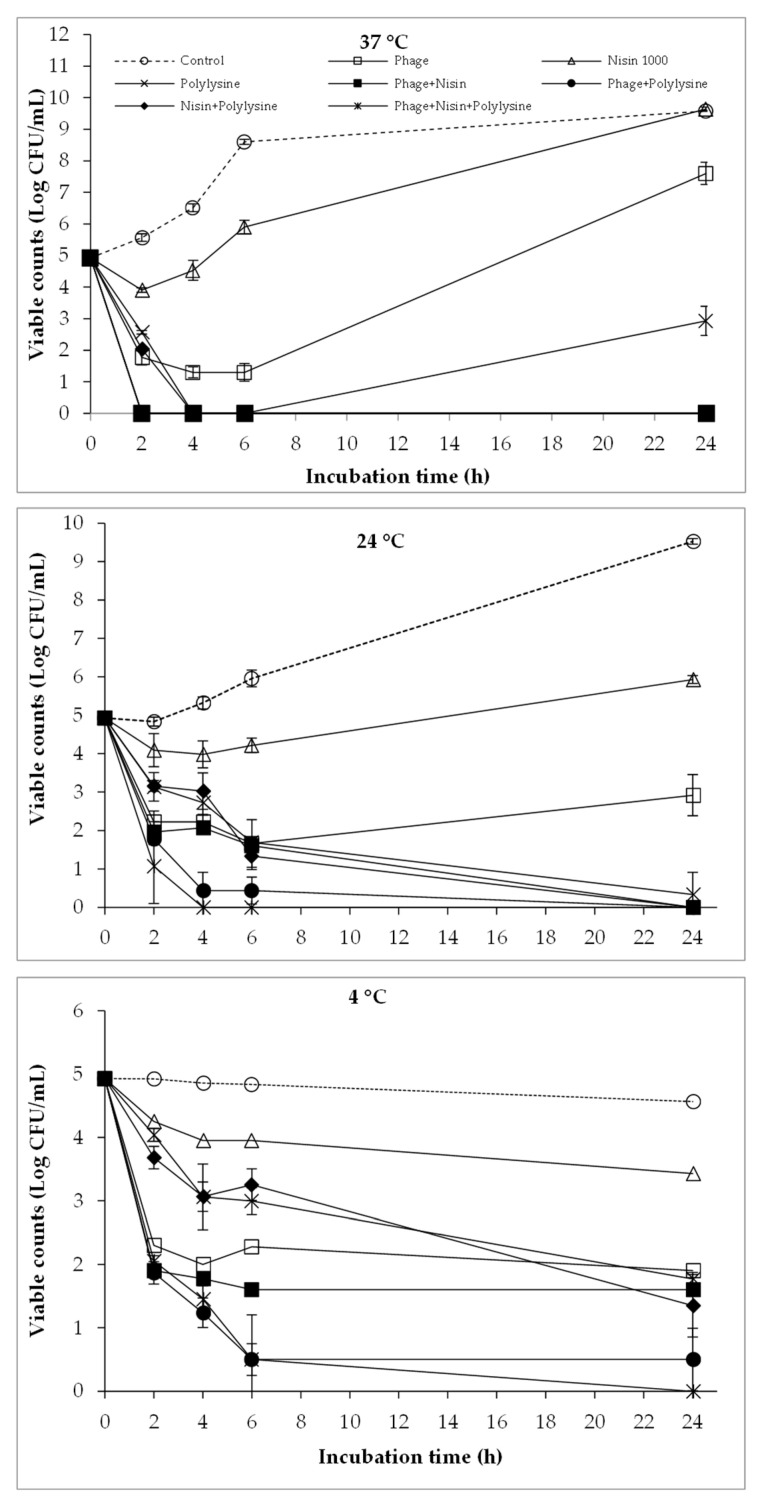
The combined effect of phages, nisin, and polylysine on bacterial viability at 37 °C, 24 °C, and 4 °C.

The combined antibacterial efficacy of phages and polylysine against bacterial cocktail is displayed in Figure 5. The combined effect of phages and polylysine was observed under all the three of the tested temperatures, and it reduced with the decreasing incubation temperature. At 37 °C, the co-treatment of phages and polylysine rapidly reduced bacterial counts to below the detection limit after 2 h, which was maintained over the whole incubation period. Although the antibacterial activity of this combination reduced at 24 °C and 4 °C, it still produced more reductions compared to the single treatment with phages or polylysine alone. The addition of nisin to the phage+polylysine combination did not result in any significant additional reduction in viable counts at 37 °C; however, it slightly lowered viable counts compared to the phage+polylysine treatment at 24 °C and 4 °C.

## 3. Discussion

Previously, the antibacterial efficacy of phages STG2, SEG5, and PS5 against their host of *S. enteritidis*, *S. typhimurium*, and *E. coli* O157:H7 has been separately evaluated in our lab, and the three phages all showed outstanding antibacterial activity [1,21]. However, bacterial regrowth was often observed several hours after phage treatment, which is regarded as a common problem in phage therapy. Even though the application of phage cocktails containing various phages might be a promising alternative, it still may not always inhibit the emergence of resistance [7]. Since bacterial resistance is difficult to control using single antibacterial agents, a combination of physical, chemical, and biological antibacterial methods, also called hurdle technology, is posited to effectively inhibit the growth of bacteria [22,23]. A variety of studies have demonstrated the effectiveness of conventional food additives, such as nisin, EDTA, and polylysine, against Gram-positive or Gram-negative bacteria due to their different damage mechanisms on bacteria [10,13,16,17,19]. Thus, the main purpose of this study is to investigate the combined efficacy of a phage cocktail in conjunction with the common food additives to control the bacterial growth and regrowth of phage-resistant populations. To the best of our knowledge, this study is the first report to highlight the significant effect of hurdle technology consisting of phages, as well as EDTA, nisin, and polylysine, against the bacterial cocktail of three important foodborne pathogens: *S. enteritidis*, *S. typhimurium*, and *E. coli* O157:H7.

In the current study, the combination of phages, EDTA, nisin, and polylysine significantly reduced the viable counts of the bacterial cocktail at various temperatures (Figure 1, Figure 2, Figure 3, Figure 4 and Figure 5), indicating the potential combined effect of the combinations. Similar hurdle effects were also reported in other studies [10,16]. Among these treatments in this study, a greater reduction in bacterial viability was observed via the co-treatment of phages with nisin or polylysine, which not only showed better antibacterial effects, but also totally inhibited the emergence of bacterial resistance during 24 h incubation at both 37 °C and 24 °C (Figure 2 and Figure 5). Similarly, the regrowth of pathogens was also not observed in the application of EDTA and nisin in combination (Figure 3). Overall, the addition of EDTA to phage+nisin produced the additional reduction in bacterial counts (Figure 4). In contrast, significant changes in viable counts were not shown by the addition of nisin to phage+polylysine at 37 °C. It could be due to the fact that phage+polylysine was strong enough to decreased bacterial viability to an undetectable level at the first time point for determination (2 h) (Figure 5). However, the addition of nisin slightly improved the antibacterial efficacy of phage+polylysine at 24 °C and 4 °C. It is well known that both EDTA and polylysine could increase the permeability of the outer membrane of Gram-negative bacteria [16,18], thus increasing bacterial susceptibility to other antibacterials, such as nisin [14], which then inhibit the further regrowth of resistant bacteria. These results are also in accordance with previous studies in which EDTA could enhance the activities of antibacterial agents against Gram-negative bacteria [13]. Explanations for the combined effect of phage and nisin that have been previously proposed are as follows: (i) phage infection and the release of phage progenies possibly accelerated when nisin formed pores in the cytoplasmic membrane of bacterial cells; (ii) bacteria may find it more difficult to repair the damage inflicted on the cell envelope caused by nisin and/or phage tail enzyme and/or lytic enzymes released during phage lysis; (iii) the multiplicity of infection (MOI) possibly increased indirectly when nisin rapidly reduced bacterial inoculum, consequently improving the effectiveness of phage, and vice versa; and (iv) phage-resistant bacterial cells were possibly killed by nisin. During the distribution and storage of food, refrigerated temperature is commonly used to maintain the fresh quality of food products [24]. Therefore, the potential antibacterial effects of combinations of phages with these food additives were also explored at 4 °C. At low temperatures, the combination of phages with nisin and polylysine exhibited great efficiency against the bacterial cocktail, even decreasing the viable counts to an undetectable level at the end of the incubation period, indicating the effectiveness of the combinations at refrigerated temperatures.

These findings indicate the potential use of combining phages and antibacterial agents at a suitable concentration to control the contamination of foodborne pathogens. This is the first study to demonstrate that the combined use of a lytic phage cocktail, nisin, EDTA, and/or polylyisne effectively inhibits the regrowth of a bacterial cocktail consisting of *S. enteritidis*, *S. typhimurium*, and *E. coli* O157:H7 during treatment. Moreover, these results also meet the demands for food distribution and storage, suggesting the potential use in the food industry to control foodborne pathogens. Further experiments are needed to evaluate the effectiveness of these combined treatments in vivo since food matrices (ionic composition, pH, fat, carbonhydrate composition, etc.) may affect the efficacy of the combination in practice.

## 4. Materials and Methods

### 4.1. Bacterial Strains, Media, and Culture Conditions

*Salmonella* Typhimurium NBRC 12,529 and *S. enteritidis* NBRC3313 used in this study were obtained from the Biological Resource Center, National Institute of Technology and Evaluation (NBRC), Chiba, Japan. Enterohemorrhagic *Escherichia coli* O157:H7 No. 196 (*stx1*^+^/*stx2*^+^) was kindly provided by the Fukuoka City Institute of Health and Environment, Fukuoka, Japan. Bacterial strains were stored in a Microbank™ (Pro-Lab Diagnostics, Richmond Hill, ON, Canada) at −80 °C. Each strain kept in Microbank™ was streaked onto Tryptic Soy Agar (TSA; Becton, Dickinson and Company, Sparks, MD, USA) and incubated at 37 °C for 24 h. A single colony was inoculated into 5 mL of Luria Bertani (LB, Becton, Dickinson and Company, Sparks, MD, USA) broth and incubated overnight at 37 °C. Prior to use, the bacterial cultures were serially diluted to obtain the desired concentrations.

### 4.2. Phage Propagation

The three lytic phages of STG2, SEG5, and PS5, specific to *S. enteritidis*, *S. typhimurium*, and *E. coli* O157:H7 used in this study, were previously isolated and characterized [1,21]. To prepare phage stock, each phage suspension was incubated with its host culture at 37 °C for 20 min. The mixture was then added to 4 mL of molten agar, poured onto TSA plates, and incubated overnight. After the incubation, 5 mL of LB was added to the top agar, and was shaken at 37 °C for 6 h. The LB and top agar containing phage particles were then collected in a centrifuge tube. The tube was centrifuged at 12,000× *g* at 4 °C for 10 min. After centrifugation, the supernatant was harvested and filtered through a membrane filter (0.22 μm pore size, Merck Millipore, Co Wicklow, Ireland). The phage titer was determined separately and the phage stock was stored at 4 °C for further use.

### 4.3. Preparation of the Phage Cocktail, Antibacterial Agents, and Bacterial Culture

Prior to each treatment, the phage (STG2, SEG5, and PS5) suspensions were mixed at equal parts to produce a phage cocktail with a titer of 10^10^ PFU/mL. EDTA (Nacalai Tesque, Kyoto, Japan), nisin (Sigma, St. Louis, MO, USA), and ε-polylysine (Chisso Corporation, Tokyo, Japan) were purchased from commercial companies and prepared at different concentrations to evaluate their combined effect with the phage cocktail (phages) [10,16]. The bacterial cultures of *S. enteritidis*, *S. typhimurium*, and *E. coli* O157:H7 were mixed at a ratio of 1:1:1 to obtain the bacterial cocktail of approximately 5 × 10^6^ CFU/mL for further use.

### 4.4. Effects of Phages and Antibaterial Agents (Alone and in Combination)

The effects of phages, EDTA, nisin, and polylisine (alone and in combination) on the viability of the bacterial cocktail consisting of *S. enteritidis*, *S. typhimurium*, and *E. coli* O157:H7 were investigated using the methods previously described by Duc, Son, and Ngan et al. (2020). Briefly, 5 mL of LB broth was inoculated with 100 µL of the bacterial cocktail to achieve the final concentration (fc) of approximately 10^5^ CFU/mL. The bacterial suspension was then treated with the phage cocktail (fc of 10^9^ PFU/mL), EDTA (fc of 0.02%), nisin (fc of 500, 1000 and 2000 IU/mL), and polylysine (fc of 0.001%) (alone and in combination). For the control, the same volume of PBS was added instead of antibacterial agents. The mixture was then incubated at various temperature conditions (37 °C, 24 °C, and 4 °C). At 2, 4, 6, and 24 h after incubation, 100 μL of the samples was withdrawn and serially diluted in PBS. The appropriate dilutions were then spread onto TSA plates and incubated overnight at 37 °C for the enumeration of viable counts.

### 4.5. Statistical Analysis

Each experiment was repeated at least three times. The results were presented as mean values and standard deviation values of the mean. Student’s *t* test (*p* < 0.05; Microsoft Excel, Mac 2016) was used to determine statistically significant differences between the treatment and control groups.

## 5. Conclusions

This study described the antibacterial effects of phage cocktail, nisin, EDTA, and polylysine (alone or in conjunction) against the bacterial cocktail consisting of *S. enteritidis*, *S. typhimurium*, and *E. coli* O157:H7 in vitro. The combination of phages with nisin or/and polysine exhibited better results than phages, nisin, or polylysine alone. Taken together, the results show the potential of combining phages with nisin and polylysine for the biocontrol of *S. enteritidis*, *S. typhimurium*, and *E. coli* O157:H7 in the food industry.

## Data Availability

The data that support the findings of this study are available from the corresponding author upon reasonable request.
